# Does the Reproductive Technique Affect Neonatal Health Parameters in Foals?

**DOI:** 10.1111/rda.70192

**Published:** 2026-03-09

**Authors:** Maria Augusta Alonso, Giovana Rodrigues dos Santos, Juliana Schleich Fonte, Pamella Costa Marques, Daniel Dantas Pereira, Fabio Luiz Buranelo Toral, Vilceu Bordignon, Jose Buratini Junior, Edward Squires, Claudia Barbosa Fernandes

**Affiliations:** ^1^ Animal Reproduction Department College of Veterinary Medicine and Animal Science – University of São Paulo São Paulo São Paulo Brazil; ^2^ In Vitro Equinos Mogi Mirim São Paulo Brazil; ^3^ Department of Animal Science Veterinary School – Federal University of Minas Gerais Belo Horizonte Minas Gerais Brazil; ^4^ Department of Animal Science, Faculty of Agricultural and Environmental Sciences McGill University Sainte‐Anne‐de‐Bellevue Quebec Canada; ^5^ Gluck Equine Research Center University of Kentucky Lexington Kentucky USA

**Keywords:** biotechnology, embryo, ICSI, neonatal, parameters, parturiations

## Abstract

This retrospective study evaluated whether different reproductive biotechnologies influence neonatal behaviour parameters in foals. Data from 102 foalings in two commercial breeding farms were analysed, including foals conceived by artificial insemination (AI), conventional embryo transfer (ET) and intracytoplasmic sperm injection (ICSI). Neonatal parameters recorded were birth weight, time to achieve sternal recumbency, time to stand, time to nurse and time to meconium elimination. Mixed statistical models were applied to assess the influence of reproductive technique, breed, sex and farm. Significant differences (*p* < 0.05) were observed between farms for time to sternal recumbency and time to nurse, and among breeds for birth height and time to meconium elimination. However, no significant differences (*p* > 0.05) were detected between sexes or among reproductive techniques for any of the parameters evaluated. Therefore, these results indicate that reproductive techniques, including advanced biotechnologies, such as ICSI, do not adversely affect neonatal health parameters in foals. On the other hand, the present findings highlight the importance of considering environmental and genetic factors, such as farm management and breed, when evaluating neonatal outcomes.

## 1 Introduction

Reproductive biotechnologies such as artificial insemination (AI), conventional embryo transfer (ET) and intracytoplasmic sperm injection (ICSI) have revolutionised equine breeding. ICSI has gained increasing global prominence for its ability to facilitate fertilisation with minimal sperm quantities and to enable precise control over both donor and recipient mares (Squires et al. 1999; Hinrichs 2018; Fonte et al. 2024; Oliveira et al. 2024).

In recent years, the use of reproductive biotechnologies has increased significantly. According to the International Embryo Technology Society (Viana 2023, 2024), the number of in vivo‐derived (IVD) equine embryos collected and transferred has declined, while the number of in vitro‐produced (IVP) embryos has risen markedly. If this trend continues, IVP is likely to surpass IVD, as the main method in equine reproduction. Only in Brazil, 22,569 IVD embryo transfers and 4110 IVP embryo transfers were performed in 2022 (Viana 2023). In 2023, these numbers increased to 24,127 and 6230, respectively (Viana 2024).

Despite these advantages, concerns persist regarding the potential impact of assisted reproductive technologies on offspring viability and health. Although such concerns remain unsubstantiated, they continue to circulate within the equine industry.

Neonatal behaviour is a critical indicator of foal viability and early adaptation, with established parameters such as time to sternal recumbency, time to stand, to nurse and meconium elimination (Magdesian 2013; Alonso et al. 2023; Halliwell and Chidlow 2023). Given the substantial costs of advanced reproductive technologies such as ICSI, establishing their safety and reliability is critical to support their broader implementation. Demonstrating the absence of abnormalities in neonatal behaviour may enhance acceptance, even among more traditional breeders.

To objectively assess early postnatal adaptation, several standardised time‐based parameters are routinely used in veterinary neonatology. These include behavioural parameters such as time to achieve sternal recumbency, time to stand, time to nurse and time to eliminate meconium. Collectively, these indicators contribute to the clinical ‘1‐2‐3 rule’ of neonatal care: the foal should stand within 1 h, nurse within 2 h, and the mare should release the placenta within 3 h (Magdesian 2013; McNaughten 2021). Deviations from this timeline may reflect compromised viability or developmental delay. Accordingly, the objective of the present study was to assess neonatal behavioural parameters in foals derived from AI, ET and ICSI reproductive biotechnologies to determine whether such techniques influence early behavioural adaptation.

## 2 Materials and Methods

### 2.1 Research Ethics

São Paulo State University, College of Veterinary Medicine and Animal Science Institutional Animal Care Committee approved all procedures described in this study (protocol No 6001260715).

### 2.2 Samples Collections

This retrospective study was conducted under field conditions, using data from two commercial breeding farms, located in the southwest of Brazil: Fazenda Santa Rita II—RAAMA (Rodovia Aldo Bolini, km 82, Piracaia—SP, ZIP code 12970‐000; Latitude: −23.030215, Longitude: −46.384757) and RH Ranch (Estrada Caixeta, S/N—Matutu, Campos dos Goytacazes—RJ, ZIP code 28175‐000; Latitude: −2.631229, Longitude: −41.269611).

**FIGURE 1:**
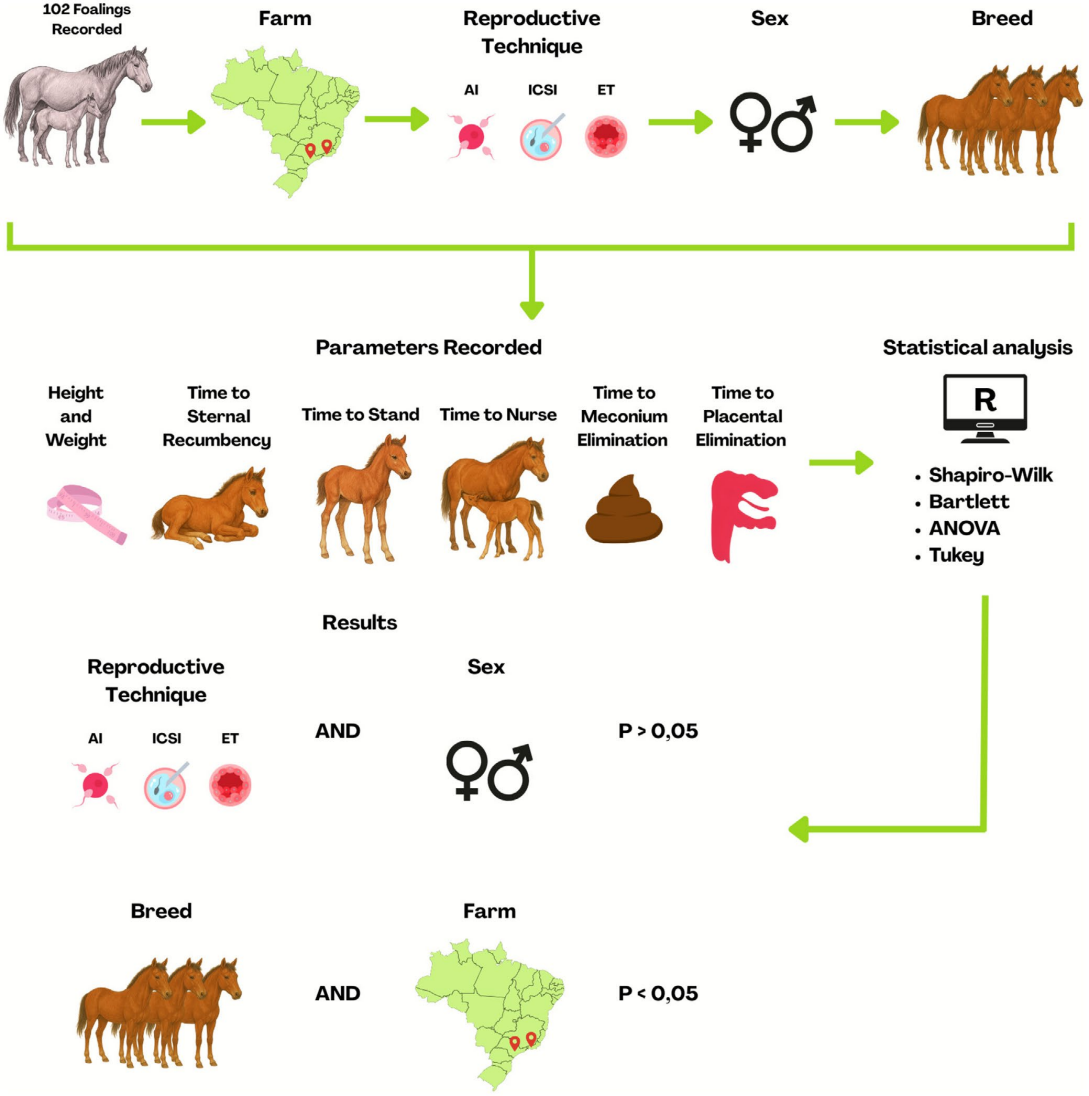
Shematic overview of the study design, recorded variables, statistical analysis and results.

All mares were kept in pasture with ad libitum access to water and mineral salt. Foals were from Brazilian Sport Horse (BSH), Mangalarga Paulista (MP) and Quarter Horse (QH) breeds. Mares were bred by AI or received a conventional ET or ICSI embryo. For AI, fresh, chilled and frozen semen were used, while conventionally embryos were transferred fresh and ICSI embryos were vitrified. Data from 102 monitored foalings across two farms were included in the study under standard management conditions with minimal human interference, enabling the assessment of neonatal outcomes under field conditions (Figure 1). As this was a retrospective analysis, not all parameters were available for every case; however, all births with sufficient data for neonatal and maternal assessments were included in the analyses. On Farm 1, these included 20 births resulting from artificial insemination (AI), 15 from intracytoplasmic sperm injection (ICSI) and 13 from embryo transfer (ET). On Farm 2, the monitored births comprised two from AI, 40 from ICSI and 12 from ET. All deliveries were supervised by a trained veterinary team to ensure accurate data collection. Among these, only three cases of dystocia were observed: one in the AI group, one in the ICSI group and one in the ET group. In all three cases, only minimal obstetrical assistance was required, and no complications occurred for either the mare or the foal.

### 2.3 Foaling Management

All mares and foals were minimally handled throughout the study. They remained in their familiar environment and were not subjected to transportation or unfamiliar procedures, thus minimising external stressors. This approach was intended to reduce the influence of handling‐related stress on physiological parameters and promoting animal welfare.

### 2.4 Perinatal Parameters

Mares were monitored throughout parturition, and the following parameters were recorded: foal height and weight, time to achieve sternal recumbency, standing, nursing and meconium passage, as well as time to placental expulsion (Figure 1). Foals were handled minimally to avoid undue interference. All data were obtained by direct observation of the foals after birth, and records were consistently collected by a trained team.

### 2.5 Statistical Analysis

This retrospective observational study analysed data from 102 foalings recorded at two commercial breeding farms in Brazil. The analysis considered the effects of reproductive technique, farm, breed and sex, as well as two‐way interactions involving sex. Due to the absence of some factor combinations in the data set, the analysis was limited to two‐way interactions with sex, restricting the assessment of higher‐order and certain other two‐way interactions.

Data normality was assessed using the Shapiro–Wilk test, and homoscedasticity was evaluated using Bartlett's test, both at a 5% significance level. The times to sternal recumbency, foal expulsion, nursing and meconium passage that did not meet these assumptions were log‐transformed [log(yᵢ + 1)], and the results were subsequently back‐transformed for presentation.

The data were analysed using a general linear model (ANOVA), considering the fixed effects reproductive technique, farm, breed and sex of the factors and their two‐way interactions. In the absence of interaction, each factor was evaluated individually. Mean comparisons were performed using Tukey's Honestly Significant Difference (HSD) test at the 5% significance level. All statistical analyses were performed using R software (version 4.2.1) (Figure 1).

## 3 Results

Overall parturition parameters of all foals included in the study are presented in Table 1. The number of observations for each parameter in each of the reproductive techniques assessed is shown in Table 2.

**TABLE 1:** Overall equine neonatal and parturition parameters.

Parameter	*N*	Min	Mean	Max	SD	CV (%)
Birth height (cm)	84	85.0	96.3	112.0	5.7	6.0
Birth weight (kg)	84	28.0	41.0	58.0	6.3	15.4
Time to sternal recumbency (min)	47	1.0	7.4	40.0	7.0	95.3
Time to stand (min)	53	10.0	35.5	71.0	14.0	39.4
Time to nurse (min)	49	20.0	84.7	157.0	37.9	44.7
Time to meconium elimination (min)	43	30.0	93.8	300.0	54.1	57.7
Time to placental elimination (min)	42	5.0	50.9	300.0	50.5	99.2

**TABLE 2:** Number of observations of equine neonatal and parturition parameters according to reproductive technique.

Parameter	Reproductive technique			Total
	AI	ICSI	ET	
Birth height	12	54	18	84
Birth weight	12	54	18	84
Time to sternal recumbency	10	26	11	47
Time to standing	11	29	13	53
Time to nursing	9	28	12	49
Time to meconium elimination	11	23	9	43
Time to placental elimination	11	21	10	42
Total	76	235	91	402

Abbreviations: AI, artificial insemination; ET, embryo transfer; ICSI, intracytoplasmic sperm injection.

Based on ANOVA (Table 3), there were no significant interactions among the variables under investigation. There was an effect of breed (Figure 1) on birth height and time to pass meconium, and an effect of farm on time to sternal recumbency and time to nurse.

**TABLE 3:** Summary of the analysis of variance (*p*‐value, coefficients of determination—*R* ^2^ and coefficient of variation—CV) for each equine neonatal and parturition parameter assessed.

Parameter	*p*							*R* ^2^	CV (%)
	Breed	Sex	Farm	Technique	Sex*breed	Sex*farm	Sex*technique		
Birth height	**0.0157**	0.8395	0.1686	0.4406	0.2091	0.7060	0.994	0.25	5.11
Birth weight	0.1540	0.9342	0.7669	0.9927	0.7696	0.5073	0.642	0.23	13.47
Time to sternal recumbency	0.2769	0.6543	**0.0012**	0.1062	0.7138	0.9687	0.257	0.60	21.20
Time to stand	0.4321	0.9534	0.4294	0.6004	0.2070	0.6689	0.100	0.20	35.02
Time to nurse	0.1040	0.8340	**0.0280**	0.8725	0.4147	0.2716	0.175	0.29	8.66
Time to meconium elimination	**0.0123**	0.3226	0.1211	0.1311	0.0696	0.7504	0.122	0.42	8.82
Time to placental elimination	0.6479	0.4264	0.8889	0.2453	0.3313	0.6633	0.103	0.30	17.28

*Note:* Values highlighted in bold indicate statistically significant differences (*p* < 0.05).

As no interactions were detected, the adjusted means are presented separately. The adjusted means for farm (Figure 1) (Table 4) indicate that more time was required for sternal recumbency and nursing at Farm 2.

**TABLE 4:** Comparison of adjusted means ± standard error (SE) in equine neonatal and parturition parameters according to farm.

Parameter	Farm		p_value
	1	2	
Birth height (cm)	97.47 ± 1.04	100.53 ± 1.72	0.1686
Birth weight (kg)	42.95 ± 1.17	42.22 ± 1.93	0.7669
Time to sternal recumbency (min)	3.56^b^ ± 0.45	9.01^a^ ± 1.82	**0.0012**
Time to stand (min)	33.50 ± 2.87	38.69 ± 5.25	0.4294
Time to nurse (min)	65.44^b^ ± 6.29	104.53^a^ ± 17.35	**0.0280**
Time to meconium elimination (min)	92.97 ± 9.22	63.55 ± 12.77	0.1211
Time to placental elimination (min)	39.26 ± 6.44	37.04 ± 12.55	0.8889

*Note:* Means followed by different letters in the same row differ significantly according to Tukey's test at the 5% significance level. Values highlighted in bold indicate statistically significant differences (*p* < 0.05).

Regarding breed, (Table 5) the mean birth height differed between BSH and QH breeds, with BSH presenting a higher mean than QH, while MP birth weight was not different in comparison to both other breeds. For time to meconium passage, BSH and MP differ significantly, with BSH showing a higher mean compared to MP, while QH was similar to BSH and MP.

**TABLE 5:** Comparison of adjusted means ± standard error (±SE) in equine neonatal and parturition according to farm.

Parameter	Breed			*p*
	BSH	MP	QH	
Birth height (cm)	102.38^a^ ± 1.77	100.25^ab^ ± 2.21	94.37^b^ ± 1.44	**0.0157**
Birth weight (kg)	45.24 ± 1.99	43.08 ± 2.48	39.43 ± 1.61	0.1540
Time to sternal recumbency (min)	4.58 ± 0.94	5.91 ± 1.53	7.00 ± 1.12	0.2769
Time to standing (min)	39.67 ± 4.99	31.00 ± 6.24	37.61 ± 4.10	0.4321
Time to nursing (min)	106.69 ± 17.45	71.46 ± 14.84	74.22 ± 9.93	0.1040
Time to meconium elimination (min)	102.47^a^ ± 18.08	48.11^b^ ± 10.58	91.97^ab^ ± 14.33	**0.0123**
Time to placental elimination (min)	41.59 ± 12.08	45.74 ± 16.64	29.11 ± 7.21	0.6479

*Note:* Means followed by different letters in the same row differ significantly according to Tukey's test at the 5% significance level. Values highlighted in bold indicate statistically differences (*p* < 0.05).

Abbreviations: BSH, Brazilian Sport Horse; MP, Mangalarga Paulista; QH, Quarter Horse.

There were no differences between gender (Figure 1) (Table 6) or among reproductive techniques (Figure 1) (Table 7) for any of the parameters assessed.

**TABLE 6:** Comparison of adjusted means ± standard error (±SE) in equine neonatal and parturition according to sex.

Parameter	Sex		*p*
	Male	Female	
Birth height (cm)	98.82 ± 1.28	99.18 ± 1.29	0.8395
Birth weight (kg)	42.67 ± 1.43	42.50 ± 1.44	0.9342
Time to sternal recumbency (min)	5.47 ± 0.87	6.05 ± 0.95	0.6543
Time to stand (min)	35.93 ± 3.85	36.25 ± 3.81	0.9534
Time to nurse (min)	84.30 ± 10.70	81.19 ± 10.20	0.8340
Time to meconium elimination (min)	85.34 ± 13.61	69.26 ± 9.20	0.3226
Time to placental elimination (min)	43.67 ± 10.33	33.29 ± 7.98	0.4264

**TABLE 7:** Comparison of adjusted means standard error (±SE) in equine neonatal and parturition according to reproductive technique.

Parameter	Reproductive technique			*p*
	AI	ICSI	ET	
Birth height (cm)	99.34 ± 1.72	97.82 ± 1.39	99.84 ± 1.44	0.4406
Birth weight (kg)	42.39 ± 1.93	42.71 ± 1.56	42.66 ± 1.62	0.9927
Time to sternal recumbency (min)	6.57 ± 1.29	6.80 ± 1.21	4.22 ± 0.80	0.1062
Time to stand (min)	40.05 ± 4.98	34.95 ± 4.39	33.28 ± 4.25	0.6004
Time to nurse (min)	85.41 ± 14.99	78.04 ± 11.19	84.95 ± 11.91	0.8725
Time to meconium elimination (min)	72.38 ± 12.40	61.75 ± 10.28	101.60 ± 18.17	0.1311
Time to placental elimination (min)	29.44 ± 8.32	54.50 ± 15.17	34.48 ± 9.07	0.2453

Abbreviations: AI, Artificial insemination; ET, embryo transfer; ICSI, intracytoplasmic sperm injection.

## 4 Discussion

The results of this study support the hypothesis that neonatal traits are not influenced by the reproductive biotechnology used to produce the foal. No significant differences were observed among the groups of foals derived from AI, ET or ICSI, nor between gender, for any of the evaluated neonatal parameters: birth weight, time to sit in sternal recumbency, time to stand, time to nurse, time to meconium elimination and time to elimination of the placenta. These findings suggest that reproductive biotechnologies, such as embryo transfer and even ICSI, do not compromise neonatal adaptation immediately after birth.

The neonatal parameters are aligned with the widely recognised ‘1‐2‐3 rule’ in equine neonatology, which states that the foal should stand within 1 h, nurse within 2 h, and the mare should expel the placenta within 3 h postpartum (Mayhew 1988; Magdesian 2013; McNaughten 2021). These parameters are critical indicators of early postnatal viability and are routinely used in both clinical and field settings to assess newborn health and adaptation. Their inclusion in this study ensures a standardised evaluation of neonatal behaviour and strengthens the clinical relevance of the findings.

Significant differences were observed between farm groups in the time to sit in sternal recumbency and to initiate nursing, with foals from Farm 2 taking longer to reach both milestones compared to those from Farm 1. This variation may reflect differences in management practices, environmental conditions, or neonatal handling protocols between facilities, underscoring the importance of standardising perinatal care when comparing outcomes across locations.

Moreover, significant differences were found among breed groups for birth height and time to meconium elimination. Variations in meconium passage time among different breeds may be explained by physiological factors, such as gastrointestinal maturity, intestinal motility and foal size at birth (Lester 2005). Larger foals, for example, tend to present more developed gastrointestinal function, which can favour earlier meconium evacuation, while smaller breeds may be predisposed to delayed elimination (Haggett and Scott 2019). These findings emphasise the need to consider breed‐specific neonatal physiology when monitoring foal health and establishing management practices.

As described by Alonso et al. (2023), in a study comparing mules and foals from mares, the time to nursing was shorter in mules. In the present study, breed‐related variations were also observed, with MP foals showing shorter times, whereas BSH foals presented longer times for both first nursing and meconium elimination. These findings support the hypothesis that nursing and meconium elimination times may be influenced by breed characteristics. To date, no studies have specifically evaluated meconium elimination time according to equine breed.

A key strength of this study lies in the large number of births/foals monitored under true field conditions, where most births occurred as uncomplicated eutocias with minimal human intervention, despite the inherent limitations of the study. Among 102 foalings, only three cases of dystocia were recorded, a substantially lower incidence than that reported by Lanci et al. (2024) in a hospital setting. This contrast emphasises the importance of assessing foals under natural, low‐stress conditions, which may influence neonatal adaptation times. Remarkably, despite differences in foaling environment, both studies report comparable behavioural outcomes, reinforcing the robustness and broad applicability of our findings.

Some methodological limitations should be acknowledged, as the retrospective design resulted in lack of control over the parameters, breed, age and parity of the mares; unequal group sizes and non‐random allocation. However, these constraints reflect real‐world breeding practices, and the study still highlights important trends that can guide future prospective research.

Finally, neonatal parameters such as time to stand, nurse and pass meconium are practical, non‐invasive indicators of early postnatal behaviour. In this study, these traits did not differ among foals produced via AI, ET or ICSI, indicating that embryo manipulation and embryo in vitro production do not impair neonatal adaptation under typical field conditions.

## 5 Conclusion

Under field conditions, neonatal behavioural parameters appear not to be influenced by reproductive biotechnologies, indicating that advanced techniques such as ET and ICSI can be applied safely in equine breeding without compromising early postnatal adaptation.

## Author Contributions

**Maria Augusta Alonso:** conceptualisation, methodology, investigation, data curation, formal analysis, writing – original draft, writing – review and editing. **Giovana Rodrigues dos Santos:** conceptualisation, methodology, investigation, writing – original draft, writing – review and editing. **Juliana Schleich Fonte:** conceptualisation, methodology, investigation, writing – original draft, writing – review and editing. **Pamella Costa Marques:** conceptualisation, methodology, investigation, writing – original draft, writing – review and editing. **Daniel Dantas Pereira:** data curation, formal analysis. **Fabio Luiz Buranelo Toral:** data curation, formal analysis, writing – original draft, writing – review and editing. **Vilceu Bordignon:** data curation, formal analysis, writing – original draft, writing – review and editing, supervision. **Jose Buratini Junior:** data curation, formal analysis, writing – original draft, writing – review and editing, supervision. **Edward Squires:** data curation, formal analysis, writing – original draft, writing – review and editing, supervision. **Claudia Barbosa Fernandes:** conceptualisation, methodology, investigation, data curation, formal analysis, writing – original draft, writing – review and editing, supervision, funding acquisition.

## Funding

This work was supported by Fundação de Amparo à Pesquisa do Estado de São Paulo, 2020/10260‐3. The authors would like to thank the owners, staff and veterinarians of Fazenda Santa Rita II and RH Ranch for their logistical support and collaboration in the data collection.

## Conflicts of Interest

The authors declare no conflicts of interest.

## Data Availability

All data supporting the findings of this study are publicly available on Google Drive at https://drive.google.com/drive/folders/1HfQTaTIaUsIdVAD‐TCnGubI‐lUdcL5Sq?usp=sharing. No restrictions apply to the availability of these data. We confirm that the data included within the manuscript have been carefully reviewed and are correctly tabulated and presented.
